# Oxidative stress induces early-onset apoptosis of vascular smooth muscle cells and neointima formation in response to injury

**DOI:** 10.1042/BSR20140122

**Published:** 2015-07-14

**Authors:** Camilo Gomez, Laisel Martinez, Annia Mesa, Juan C. Duque, Luis A. Escobar, Si M. Pham, Roberto I. Vazquez-Padron

**Affiliations:** * Department of Surgery and Vascular Biology Institute, University of Miami Miller School of Medicine, Miami, FL 33136, U.S.A.; † Department of Surgery, University of Maryland School of Medicine, Baltimore, MD 21201, U.S.A.

**Keywords:** apoptosis, MAPK, NAD(P)H oxidase inhibitor, remodelling, superoxide dismutase, vascular injury

## Abstract

The present study dissects the mechanisms underlying the rapid onset of apoptosis that precedes post injury vascular remodelling. Using the rat balloon injury model, we demonstrated that a significant number of arterial vascular smooth muscle cells (VSMC) undergo apoptosis at 90 min after the procedure. This apoptotic wave caused significant loss in media cellularity (>90%) over the next 3 h and was accompanied by a marked accumulation of oxidative stress by-products in the vascular wall. Early apoptotic VSMC were rich in p38 mitogen-activated protein kinase (MAPK) and the transcription factor c-Jun and secreted IL-6 and GRO/KC into the milieu as determined using multiplex bead assays. Neointima thickness increased steadily starting on day 3 as a result of pronounced repopulation of the media. A second apoptotic wave that was detected at 14 days after injury affected mostly the neointima and was insufficient to control hyperplasia. Suppression of reactive oxygen species (ROS) production using either the NAD(P)H oxidase inhibitor VAS2870 or pegylated superoxide dismutase (PEG-SOD) significantly decreased the number of apoptotic cells during the first apoptotic wave and showed a trend towards reduction in the neointima-to-media thickness ratio at 30 days post injury. These results indicate that oxidative stress in response to injury induces early-onset apoptosis of VSMC through the activation of redox-sensible MAPK pro-apoptotic pathways. This remodelling process leads to the local accumulation of inflammatory cytokines and repopulation of the media, which ultimately contribute to neointima formation.

## Abstract (precis)

The rapid onset of VSMC apoptosis after arterial injury is driven by the accumulation of reactive oxygen species in the vascular wall and the activation of redox-sensible MAPK pathways. This process leads to vascular inflammation and neointimal hyperplasia.

## INTRODUCTION

The adaptive response of the vascular wall to mechanical injury determines the outcome of endovascular interventions. This response is characterized by vascular smooth muscle cells (VSMC) apoptosis [1–3], inflammation [4] and cell proliferation [3,5] that when unbalanced may lead to adverse wall remodelling and neointima formation. Despite being an area of active research, the molecular mechanisms underlying each of these processes or how they interrelate to each other have not been fully identified. This incomplete understanding poses a challenge for the prevention of post-angioplasty complications.

Although very low levels of cell turnover are detected in normal adult arteries [6], apoptosis and proliferation of VSMC are important, ongoing processes in vascular remodelling [7–9]. Consequently, inadequate levels of VSMC apoptosis have been associated with rupture of aneurysms [10], instability of atherosclerotic plaques [11,12] and restenosis [13,14]. In the setting of arterial injury, too little apoptosis can lead to excessive VSMC proliferation and neointima growth [15], whereas too much can result in impaired apoptotic clearance leading to secondary necrosis and inflammation [8,9].

Two waves of VSMC apoptosis are frequently detected in injured arteries. The first one is referred to as ‘early-onset apoptosis’ and occurs in the media within 30 min to 6 h after injury, depending on the animal model [1–3]. The second wave or ‘late apoptosis’ is sometimes detected days or weeks later in the neointima, and to a lesser extent in the media, and is associated with chronic remodelling to limit neointimal hyperplasia (NIH) [1,5,16]. The role of early-onset apoptosis in arterial reocclusion as a result of NIH is still debatable, as it may depend on the magnitude of the angioplastic injury [15]. Some studies suggest that low levels of apoptosis after vascular injury lead to restenosis [14,15], whereas others show that massive apoptosis triggers VSMC proliferation and precedes NIH [3,5,13]. Unfortunately, very few studies have looked at the molecular mechanisms driving this process in their respective models [1,17], which limits the extent of these comparisons.

Our work in the rat iliac injury model demonstrates that early-onset apoptosis after balloon injury is induced by oxidative stress and activation of redox-sensible mitogen-activated protein kinase (MAPK) pro-apoptotic pathways. This process leads to neointima formation starting on day 3 after injury, likely as a result of inflammation from incomplete clearance of oxidative products and VSMC migration from the proliferating media. Both maximal medial VSMC repopulation and a late apoptotic wave affecting the neointima coincide at 14 days after injury, indicating an active remodelling process that is ultimately insufficient to control NIH. Lastly, suppression of reactive oxygen species (ROS) production using either a NAD(P)H oxidase inhibitor or superoxide dismutase significantly decreases early-onset apoptosis of VSMC, with a noticeable effect in the reduction in neointima formation.

## MATERIALS AND METHODS

### Balloon injury model

Fischer 344 rats (2–4 month-old) were obtained from Harlan Laboratories. The balloon injury was inflicted in the right iliac artery with a 2F Fogarty catheter (Baxter) adapted to a custom angiographic kit (Boston Scientific, Scimed) as previously described [18]. Arterial specimens were collected at 10, 30, 60 and 90 min, 3 h and 1, 3, 7, 14 and 30 days after injury from formalin-perfused fixed animals [19]. Alternatively, animals were perfused with PBS and arterial tissue was snap-frozen for protein analysis. The contralateral iliac artery was used as non-injured control. The Institutional Animal Care and Use Committee at the University of Miami had previously approved all studies.

### Inhibition of ROS production

The NAD(P)H oxidase inhibitor VAS2870 and pegylated superoxide dismutase (PEG-SOD) were obtained from Sigma–Aldrich and dissolved in 20% Pluronic F-127 (Sigma–Aldrich) at 250 μg/ml and 1667 units/ml concentrations, respectively. Both solutions were prepared and maintained at 4°C until use. Prior to injury, 300 μl of dissolved VAS2870, PEG-SOD or vehicle were applied perivascularly to the artery and allowed to solidify for 30 min, after which balloon injury was performed. Arterial specimens were collected at 90 min and 30 days post injury for analysis.

### Histology and morphometric analysis

Arteries were paraffin-embedded and sectioned at American Histolabs. The neointimal area was calculated from haematoxylin and eosin stained slides using Image Pro Plus (Media Cybernetics) [20].

### Apoptotic VSMC detection

Apoptosis was detected in paraffin sections using the ApopTag Peroxidase *In Situ* Oligo Ligation Kit (Millipore) according to the manufacturer's guidelines with some modifications [20]. After developing colour in peroxidase substrate for detection of apoptotic nuclei, sections were incubated with the anti-smooth muscle actin (SMA) antibody clone 1A4 (1:400; DAKO) for 1 h at RT. The Envision G/2 System/AP (permanent red) kit (DAKO) was used to visualize VSMC. Apoptotic cells were identified by a dark brown nucleus surrounded by red cytoplasm. The percentage of positively stained apoptotic cells was determined by randomly counting 10 fields per section.

For confirmation of apoptosis by transmission electron microscopy 30 min after injury, arteries were excised and fixed in 2.5% glutaraldehyde, 4% paraformaldehyde and 0.1 M sodium cacodylate. Sections were post-fixed in 1% osmium tetroxide, dehydrated, stained en bloc with 3% uranyl acetate and embedded in epoxy resin. Thin sections were examined with a Philips 300 electron microscope.

### Immunohistochemistry

After section rehydration, endogenous peroxidase was blocked with 3% hydrogen peroxide. Epitope retrieval was performed as previously described by boiling slides in citrate buffer [21]. Nonspecific binding was blocked with 0.5% blocking solution (DAKO). Slides were incubated with primary antibodies for 1 h at room temperature. Biotinylated secondary antibodies (DAKO Universal Link) were applied for 30 min, followed by a washing step with PBS and 15-min incubation with horseradish peroxidase–streptavidin solution (DAKO) at room temperature. Colour was developed with a DAB chromogenic solution (DAKO). Nuclei were counterstained with Meyer's haematoxylin and mounted as described above. Primary antibodies were directed against 8-hydroxydeoxyguanosine (Millipore), 3-nitrotyrosine (Millipore) and advanced glycation end products (AGEs) (Abcam).

### Cytokine assays and phosphoproteins

Estimation of vascular concentrations for 13 cytokines and chemokines was performed using a LINCOplex Rat Cytokine Assay (24-Plex; EMD Millipore) [20]. Phosphoproteins were measured in injured and control arteries using the Beadlyte 8-plex multi-pathway signaling kit (EMD Millipore). Briefly, 100 μg of protein lysate were loaded into wells pre-coated with colour-coded antibody-immobilized beads. Specific proteins bound to fluorescent beads were detected using a mix of protein-specific, biotinylated detector antibodies followed by incubation with streptavidin–PE. Multiplex were resolved in a Bio-Plex 100 (Bio-Rad) bead sorter. Values were expressed as fold of non-injured arteries.

### Statistical analyses

Results were expressed as means ± S.E.M. Two-group comparisons were conducted using two-tailed *t*-tests for independent samples with unequal variances. Statistics were calculated with Prism 5 (GraphPad Software).

## RESULTS

### Early-onset apoptosis targets medial VSMCs and induces remodelling

We used the rat iliac balloon angioplasty model to investigate the mechanisms leading to post injury VSMC apoptosis and its effects on wall remodelling [18]. Haematoxylin and eosin staining revealed abundant condensation and cytoplasmic shrinkage in VSMC of injured walls harvested 90 min after surgery compared to the contralateral non-injured controls (Figures 1A and 1B). These cells were apoptotic (24.7±3.4%; Figure 1H) as demonstrated by *In Situ* Oligo Ligation staining and expression of activated caspase 3 (Figures 1E and 1G). Further characterization of apoptotic cell morphology by transmission electron microscopy confirmed cytoplasmic shrinkage, nuclear condensation and disintegration, and formation of apoptotic bodies (Figures 1J and 1K). In contrast, VSMC in sections of non-injured control vessels stained negatively for apoptotic markers (Figures 1D and 1F), consistent with the low level of cell turnover in healthy vessels [6]. This first wave of apoptosis led to a 90% decrease in media cellularity over the first 3 h (Figure 1C).

**Figure 1:**
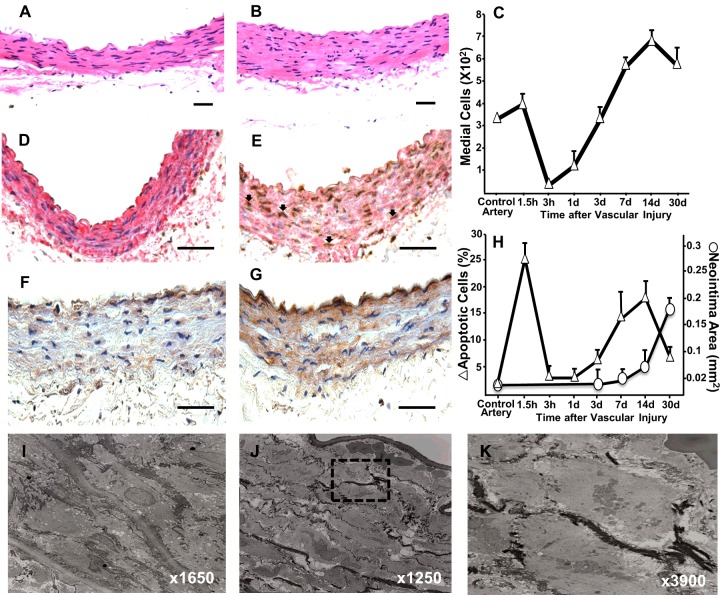
Early-onset apoptosis precedes vascular remodelling after arterial injury in rats (**A** and **B**) Haematoxylin and eosin stained cross sections from control (**A**) and injured (**B**) iliac arteries harvested at 90 min after surgery. (**C**) Temporal changes in the number of medial VSMC after vascular injury. (**D** and **E**) Detection of apoptotic VSMC in control (**D**) and injured (**E**) arteries at 90 min using a combination of *In Situ* Oligo Ligation (brown) to detect apoptotic nuclei and immunohistochemistry for SMA (red). Representative apoptotic VSMC are shown by arrows. (**F** and **G**) Representative microphotographs of control (**F**) and injured (**G**) arteries harvested at 90 min after surgery and stained for activated caspase 3. (**H**) Temporal quantification of apoptotic cells and neointima formation in the rat injured artery. Each point represents the mean ± S.E.M. (*n*=5-7). (**I**–**K**) Transmission electron microscopy of a normal (**I**) and injured artery (**J**). Apoptotic nuclei in the box are magnified in panel **K**. Scale bars=50 μm.

A time-course assessment of apoptotic VSMC in injured vessels over 30 days after the procedure indicated the occurrence of two apoptotic waves (Figure 1H), although only the first one affected the media (Figure 1C). Media repopulation started at 3 h after injury and reached approximately 200% cellularity compared with control arteries at 14 days (Figure 1C). Neointima formation was visible at 7 days (Figure 1H), likely as a result of VSMC migration from the media. The second apoptotic wave was less pronounced than the first one (18.3±3.6%) and insufficient to prevent overt NIH at 30 days post injury (Figure 1H).

### Early apoptosis is driven by oxidative stress and activation of redox-sensible MAPK pathways

Carotid artery injury increased vascular ROS production in rats [22]. In addition, oxidative stress has been associated with angioplasty-induced medial cell apoptosis in rabbits [17]. Therefore, we turned to investigate whether similar stimuli contributed to remodelling in the rat iliac injury model. To this end, immunohistochemistry was used to screen for oxidative byproducts in the vascular wall of injured and non-injured arteries (Figure 2). Accumulation of 3-nitrotyrosine (Figures 2A–2E), AGEs (Figures 2F–2J) and 8-oxo-2′-deoxyguanosine (8-oxo-dG) (Figures 2K–2O) was visible in injured arteries starting at 30 min after surgery, but not in control vessels (Figures 2A, 2F, 2K). For all three, maximal vascular concentration occurred at 90 min post injury and most cleared by 3 h, with the exception of AGEs, which remained elevated at 24 h (Figure 2J). This timeline coincided with the peak of early-onset apoptosis, as indicated in Figure 1H. The three byproducts tested are markers of cell damage in atherosclerosis and other pathological conditions [23–25]. AGEs are glycated proteins or lipids that participate in the production of ROS [25], 3-nitrotyrosine is a product of aromatic amino acid nitration by ROS [24] and 8-oxo-dG is a marker of oxidative DNA damage [23].

**Figure 2:**
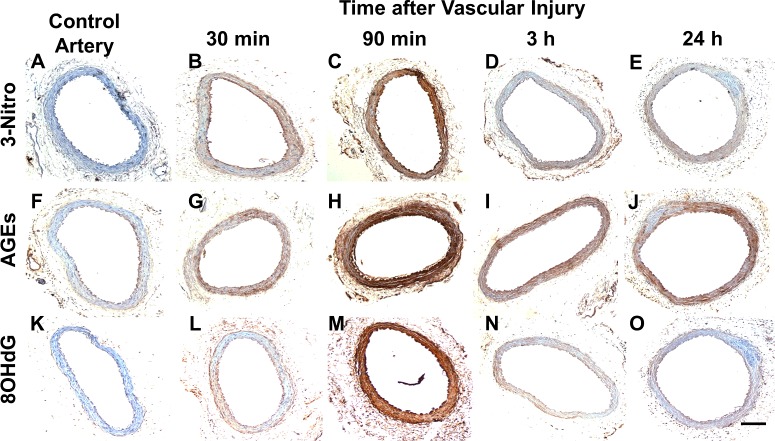
Oxidative byproducts rapidly accumulate in vascular tissue after arterial injury Cross sections from control (**A**, **F**, **K**) and injured arteries harvested at 30 min (**B**, **G**, **L**), 90 min (**C**, **H**, **M**), 3 h (**D**, **I**, **N**) and 24 h (**E**, **J**, **O**) after surgery were stained for 3-nitrotyrosine (**A**–**E**), AGEs (**F**–**J**) and 8-hydroxy-2′-deoxyguanosine (**K**–**O**). Staining is noticed in brown with nuclei counterstained with haematoxylin. Scale bar=100 μm.

Further characterization of the cellular response to oxidative stress led us to investigate the activation of redox-sensible MAPK pro-apoptotic pathways. P38 MAPK and c-Jun N-terminal kinase (JNK) are serine/threonine kinases that get activated by upstream phosphorylation cascades in response to stress stimuli, including oxidation [26]. They, in turn, phosphorylate multiple cellular targets and modulate expression of genes that participate in induction of apoptosis and inflammation [26]. Quantification of phosphorylated p38 MAPK and JNK's downstream transcription factor c-Jun using a multiplex phosphoprotein assay demonstrated that they are both elevated during early-onset apoptosis in injured arteries compared with control vessels (Figures 3A and 3B). The level of activated p38 MAPK at 30 min post injury was 20 times higher in injured compared with control arteries (Figure 3A), and this occurs prior to the early apoptotic peak of VSMC in the media (Figure 1H). Similarly, the level of phosphorylated c-Jun was approximately 2.5 times higher in injured arteries compared with controls from 1 to 1.5 h after surgery (Figure 3B), when medial cell loss was at its maximum. Activation of c-Jun at a later time point than p38 MAPK can be explained by its further downstream position in the JNK pathway. The levels of other phosphoproteins (i.e., Akt, ERK1, HSP27, MEK, NF-kB and p70S6K) did not change over the first 24 h after injury between treated and non-treated arteries (data not shown), indicating that they are not associated with early VSMC apoptosis.

**Figure 3:**
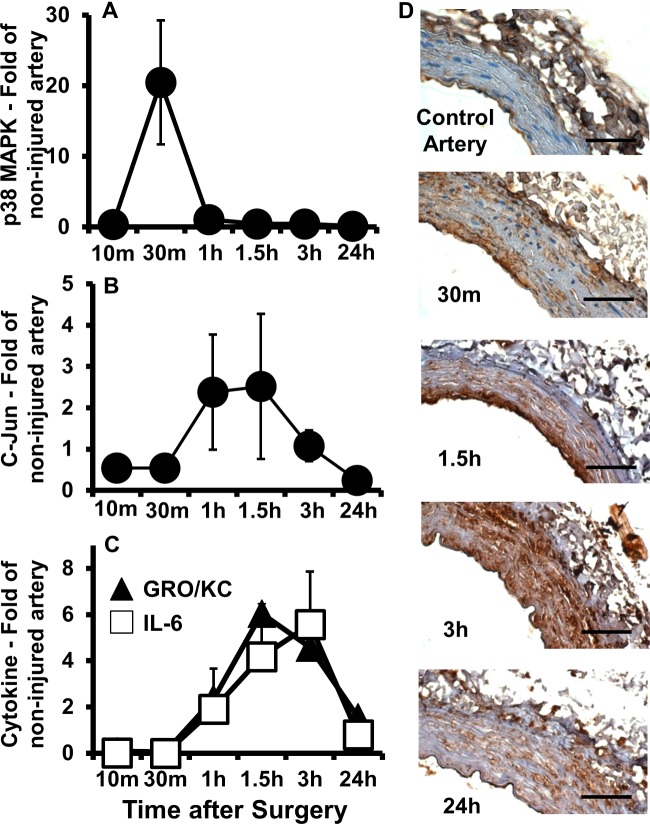
Quick activation of MAPK pathways and temporal accumulation of vascular cytokines in injured compared with control arteries (**A**–**C**) Levels of phosphorylated p38 MAPK (**A**), phosphorylated c-Jun (**B**) and the inflammatory cytokines IL-6 and GRO/KC (**C**) over time in injured compared with non-injured contralateral arteries as determined using multiplex immunoassays. Each point represents the mean ± S.E.M. (*n*=5). (**D**) Representative immunohistochemistry cross sections stained for IL-6 in control and injured arteries harvested at 30 min, 1.5 h, 3 h and 24 h after balloon injury. Scale bar=50 μm.

### Early-onset apoptosis leads to vascular accumulation of inflammatory cytokines

Since oxidative byproducts are not completely cleared from the wall by 24 h post injury (Figures 2E, 2J and 2O) and p38 MAPK and JNK are also involved in inflammation [26], vascular concentrations of 13 cytokines and chemokines were measured after balloon injury in treated compared with control arteries. The 13 factors include eotaxin, GRO/KC, IFNγ, IL-1β, IL-2, IL-6, IL-9, IL-10, IL-18, leptin, MCP-1, TNFα and VEGF. From this battery of cytokines and chemokines, only GRO/KC and IL-6 increased significantly in injured arteries compared with controls (Figure 3C), suggesting that they are a signature of early-onset apoptosis after arterial injury. The vascular levels for the remaining factors did not change significantly over the time tested (data not shown). Both IL-6 and GRO/KC first appeared elevated at 1 h post injury, reached levels 4–6-fold higher than non-injured arteries at 1.5–3 h, and remained high until the 24 h time point (Figure 3C). Immunohistochemistry staining for IL-6 in arterial cross sections confirmed that it localized almost exclusively in the media of injured arteries, whereas no reactivity was detected in non-injured controls (Figure 3D). These results indicate that early-onset apoptosis induces inflammation in the vascular wall, which likely contributes to remodelling after injury.

### Inhibition of ROS production reduces early-onset apoptosis and neointima formation

In order to confirm the predominant role of ROS production in vascular remodelling after injury, antioxidant therapies or vehicle were applied perivascularly 30 min prior to the balloon procedure. Pre-treatment with either the NAD(P)H oxidase inhibitor VAS2870 or PEG-SOD resulted in a significant reduction in apoptotic VSMC at 90 min post injury compared with the vehicle control (5.25±2.50, 4.00±1.63 and 132.00±24.83 cells per high power field, respectively, *P* =  0.002 for both comparisons; Figures 4A–4D). In addition, although not statistically significant, there was a noticeable decrease in the neointima-to-media thickness ratio at 30 days by both treatments compared with control (0.13±0.09 for VAS2870, 0.08±0.04 for PEG-SOD and 0.26±0.08 for vehicle; Figure 4E). This trend towards NIH reduction was most statistically evident with PEG-SOD (*P* =  0.07 compared with vehicle). These results demonstrated that regulation of ROS production through antioxidant therapies prevents early-onset apoptosis of VSMC and shows a trend towards attenuation of NIH.

**Figure 4:**
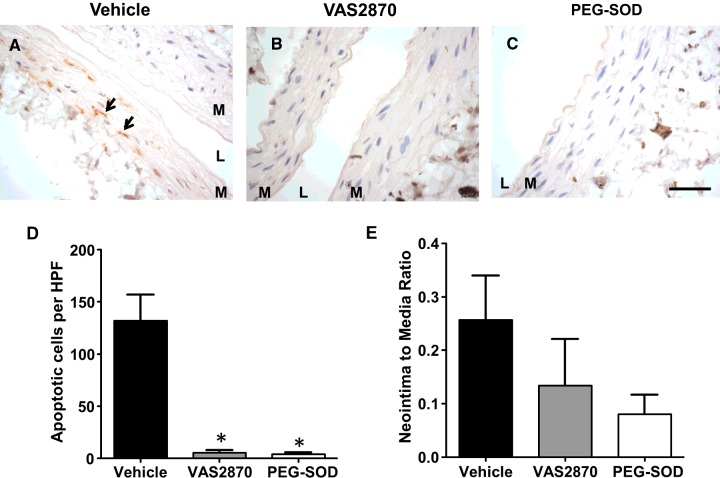
Perivascular pre-treatment with antioxidants significantly reduces early-onset apoptosis and shows a trend towards attenuation of NIH (**A**–**C**) Detection of apoptotic VSMC at 90 min post injury in arteries that were pre-treated with either vehicle (**A**), VAS2870 (**B**) or PEG-SOD (**C**) using *In Situ* Oligo Ligation (brown) to detect apoptotic nuclei. Representative apoptotic VSMC are shown by arrows. Scale bar=30 μm. (**D**) Quantification of apoptotic cells per high power field (HPF) in injured arteries pre-treated with antioxidants or vehicle and harvested at 90 min post injury. Values are represented as the mean ± S.E.M. (*n*=3–4), *P*< 0.05. (**E**) Neointima-to-media thickness ratio at 30 days post injury in arteries that were pre-treated with antioxidants or vehicle. Values are represented as the mean ± S.E.M. (*n*=4–6).

## DISCUSSION

Restenosis secondary to NIH is a major cause of failure of endovascular surgical interventions [27]. Although second generation drug-eluting stents have greatly reduced adverse events and need for revascularization procedures after percutaneous coronary interventions [28,29], arterial reocclusion continues to be a problem with other types of angioplasty procedures [27]. In addition, drug-eluting stents do not come without risks, have increased costs and are not suitable for all clinical situations [27]. Therefore, understanding the mechanisms that drive negative remodelling after arterial injury can have important implications for the design of treatments that will benefit a higher number of angioplasty patients.

Our work demonstrates that oxidative stress induces early-onset apoptosis after arterial injury, which leads to adverse wall remodelling as a result of excessive media repopulation and neointima formation. Quick accumulation of ROS in the wall and activation of the p38 MAPK and JNK pathways drive the first apoptotic wave and contribute to vascular inflammation and NIH. In addition, inhibition of ROS production using either a NAD(P)H oxidase inhibitor or PEG-SOD prevents early-onset apoptosis of VSMC and shows a noticeable reduction in NIH. Our results are in agreement with previous studies indicating that antioxidant therapies modulate vascular remodelling after injury [17,22,30,31], although different mechanisms have been proposed depending on the animal model. Early apoptosis of medial cells in the rabbit balloon injury model was reduced by administration of *N*-acetyl cysteine or pyrrolidine dithiocarbamate, and this effect was mediated through inhibition of the JNK pathway [17]. In contrast, Chen et al. [30] postulated that reduction in NIH by hydrogen-rich saline administration in the rat balloon injury model occurred through inactivation of ERK1/2 and Akt. In the latter case, identification of the pathways was done by testing arterial samples at 14 d after injury, well past early-onset apoptosis and by studying the effects of the treatment on fetal bovine serum (FBS)-induced proliferation and ROS production in culture [30]. In contrast, our work did not show activation of Akt or ERK1/2 in early apoptosis after injury. This difference can perhaps be explained by the undergoing VSMC proliferation observed by Chen et al. at 14 days in injured arteries, which might be driven by these enzymes. Moreover, unlike ERK1/2, p38 MAPK and JNK are only weakly activated by growth factors [26]; therefore, analysis of NIH reduction by hydrogen-rich saline might yield a different pathway if assessed at an earlier time point and using a non-FBS supplemented methodology.

The drop in glutathione levels in rabbit arteries after angioplastic injury followed by medial cell apoptosis [17] suggests that VSMC are not capable of overcoming the oxidative stress induced by the procedure. In fact, our data show incomplete clearance of oxidative byproducts at 24 h after surgery, in particular AGEs. This environment leads to further inflammation, as reflected by concurrent accumulation of IL-6 in the media at the time of peak concentrations of oxidative byproducts. Interestingly, induction of VSMC proliferation and migration *in vitro* by apoptotic VSMC supernatants is obliterated by IL-6 inhibition [7], indicating that this cytokine is secreted by apoptotic VSMC and crucial for media repopulation and neointima formation. High IL-6 concentrations in coronary sinus blood have also been associated with increased risk of late restenosis after percutaneous coronary interventions [32].

Overall, our work demonstrates for the first time that oxidative stress-mediated activation of JNK and p38 MAPK is responsible for early-onset apoptosis of VSMC after arterial injury in rats. More importantly, our results show that regulation of ROS production through antioxidant therapies not only prevents the massive apoptosis of medial cells but also shows a trend towards attenuation of subsequent NIH. Our choice of antioxidants revealed that NAD(P)H oxidase is involved in the generation of ROS after balloon injury. Considering that ROS play an important pathological role in atherosclerosis and that both kinase pathways have been shown to coordinate cellular responses to NAD(P)H oxidase, a major ROS-producing enzyme in VSMC [33,34], an investigation of these pathways in atherosclerotic human arteries before and after angioplasty is warranted.

## Abbreviations

AGE: advanced glycation end product
JNK: c-Jun N-terminal kinase
MAPK: mitogen-activated protein kinase
NIH: neointimal hyperplasia
8-oxo-dG: 8-oxo-2′-deoxyguanosine
PEG-SOD: pegylated superoxide dismutase
ROS: reactive oxygen species
SMA: smooth muscle actin
VSMC: vascular smooth muscle cells
